# Global trends in the health economics field of PD-1/PD-L1 inhibitors: A bibliometric and visualized study

**DOI:** 10.3389/fphar.2023.1141075

**Published:** 2023-03-22

**Authors:** Sicen Lai, Licong Xu, Liang Zhang, Lanyuan Peng, Yixin Li, Yuancheng Liu, Nianzhou Yu, Wangqing Chen, Kai Huang

**Affiliations:** ^1^ Department of Dermatology, Xiangya Hospital, Central South University, Changsha, China; ^2^ Hunan Engineering Research Center of Skin Health and Disease, Central South University, Changsha, China; ^3^ Hunan Key Laboratory of Skin Cancer and Psoriasis, Xiangya Hospital, Central South University, Changsha, China; ^4^ XiangYa School of Medicine, Central South University, Changsha, China; ^5^ National Clinical Research Center for Geriatric Disorders, Xiangya Hospital, Central South University, Changsha, China; ^6^ Department of Dermatology, Wuhan No. 1 Hospital, Wuhan, China

**Keywords:** PD-1, PD-L1, the health economic, cancer immunotherapy, bibliometric analysis, future trends

## Abstract

Inhibitors of programmed cell death protein 1 and its associated ligand (PD-L1) are widely used in cancer treatment. However, medical costs and benefits of PD-1/PD-L1 inhibitors need attention owing to differences in response rates among individuals. This study explored global trends in the health economics field of PD-1/PD-L1 inhibitors to enhance their worldwide development. Bibliometric analysis of all documents currently indexed in Web of Science Core Collection from inception to 2022 was performed. Publication year, authors, countries, institutes, and journals were analyzed by Bibliometrix package (version 3.2.1) in R (version 4.1.3). CiteSpace (version 6.1.R6) and VOSviewer (version 1.6.18) were used to analyze burst words, co-authorship of institutes, co-cited journals, and co-cited references, while figures were mainly drawn by Ggplot2 package (version 3.3.5) in R (version 4.1.3) and SCImago Graphica Beta (version 1.0.23). A total of 2020 documents related to the health economics of PD-1/PD-L1 inhibitors were identified, and 1,204 documents met the selection criteria for inclusion in the study. A rapid increase in the number of publications since 2019 was observed, but this increase stopped in 2022, revealing research saturation in the field. *Value in Health* (166 publications, 13.79% of total documents) had the most publications, while *New England Journal of Medicine* (2,890 co-citations) was the most co-cited journal. The United States was the leading contributor in this field with 506 publications and the top two productive institutes globally. The main hot topics included the cost-effectiveness of treatment with PD-1 and/or PD-L1 inhibitors, and the comparison between the cost-effectiveness of PD-/PD-L1 inhibitors and other drugs. There were substantial differences between developed and developing countries in the health economics field of PD-1 and/or PD-L1 inhibitors. The cost-effectiveness analysis of combined treatment with PD-1/PD-L1 inhibitors and other drugs warrants further attention. Findings from this study may provide governments and pharmaceutical companies with a strong reference for future research.

## Introduction

Cancer is one of the top three causes of death before the age of 70 years in 177 countries according to the World Health Organization (Bray et al., 2021; World Health Organization, 2022). Inhibitors of programmed cell death protein 1 (PD-1) and its associated ligand (PD-L1) as medicines of immunotherapy, the most promising treatment for cancers (Tang J, 2018; Cancer Research Institute, 2022), have been used for non-small-cell lung cancer (Brahmer et al., 2015; Jia et al., 2018; Gao et al., 2019), Hodgkin’s lymphoma (Ansell et al., 2015; Jia et al., 2018; Gao et al., 2019), and others in recent years. However, some disadvantages of PD-1/PD-L1 inhibitors have emerged, such as marked differences in effective response rate among different people (Su et al., 2019; Daassi et al., 2020; Beaver and Pazdur, 2022) and the high cost of the inhibitors (Tarhini et al., 2019; Cheng et al., 2022).

Use of PD-1/PD-L1 inhibitors is uneven worldwide. Attested by Frost and Sullivan in 2019 (Frost & Sullivan, 2022), the size of the global market for PD-1/PD-L1 inhibitors exceeded $16 billion in 2018, with the Chinese and North American markets accounting for approximately 6.25% and 60% of the global market size, respectively. Factors confining the development of health economics research of PD-1/PD-L1 inhibitors include, but are not limited to, the time research commenced, the efficiency of the development, etc. (Wagstaff and Culyer, 2012). Thus, starting the experiment earlier and developing the drugs faster results in larger ownership of the markets. For instance, as early as 2015, the United States, who occupies the largest share of the global market for PD-1 inhibitors and PD-L1 inhibitors, first published a document about the health economics of Pembrolizumab after finishing clinical trials for the drug and starting to use it for the treatment of the metastatic melanoma (Tan and Quintal, 2015). Furthermore, six kinds of PD-1 inhibitors and PD-L1 inhibitors developed by the United States were in use in different countries before 2021 (National Medical Products Administration, 2022; U.S. Food & Drug Administration, 2022). Research in health economics can help in clinical drug selection to maximize patient benefit and can also help develop an optimization strategey suitable for the market and clinical practice of PD-1/PD-L1 inhibitors (Wagstaff and Culyer, 2012; Husereau et al., 2022). Therefore, research on the health economics of PD-1/PD-L1 inhibitors is essential.

Bibliometrics may be a prominent approach to reveal the research status and development trend of health economics on PD-1/PDL-1 inhibitors by visualization and statistical analysis. Previous bibliometric analysis was conducted to research the PD-1/PD-L1 inhibitors in the cancer field (Cancer Research Institute, 2022). However, there is currently an absence of bibliometric research on the health economics of PD-1 and PD-L1 inhibitors. Thus, in this study, a bibliometric analysis was conducted to 1) comprehensively understand the global trend of health economics of PD-1 inhibitors and PD-L1 inhibitors; 2) make suggestions for the future development of PD-1 inhibitors and PD-L1 inhibitors by pharmaceutical companies; 3) and help developing and developed countries to improve care to reduce the burden of patients’ treatment.

## Materials and methods

### Search strategy and criteria

**FIGURE 1:**
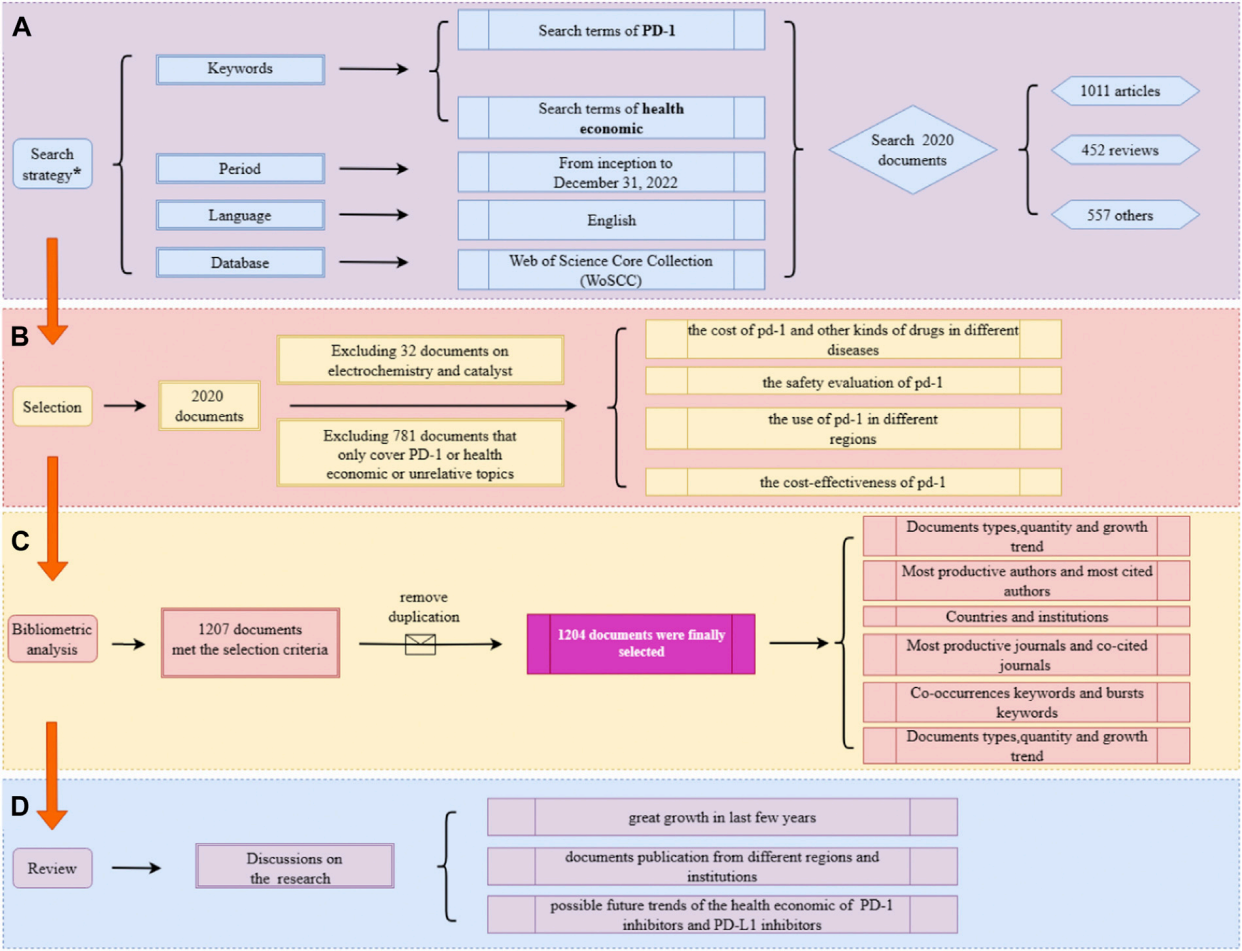
Overview of the study flow diagram. **(A)** The search strategy. **(B)** The selection process. **(C)** The process of bibliometric analysis. **(D)** Results of the analysis.

The Web of Science Core Collection database was fully searched from its inception to 31 December 2022. The search query included two main terms: “PD-1” and “the health economic”. An additional file shows more details about the search strategy. There were no restrictions on the language, document type, data category, or year of publication. Subsequently, a preliminary selection was completed by the title, keywords, and the abstract of the document. For records that could not be clearly judged by reading these three components, a secondary screening was conducted by reading the content of the identified records. The selection of records and flow chart of the research framework are shown in Figure 1. In addition, a more detailed searching strategy is demonstrated in Supplementary Table S1.

### Analysis method

The preliminary search found 2020 documents, which were then further analyzed to select those suitable for inclusion in the study. First, 32 documents relating to electrochemistry research, including some documents about catalysts like palladium (Pd), were excluded, then excluded 781 documents that only covered topics about economics or PD-1/PD-L1, which referred to two types of documents unrelated to the research, one is documents that analyzed economic effectiveness but the objects of documents were not PD-1/PD-L1 inhibitors; another type is documents working on PD-1/PD-L1 inhibitors, but the research content had nothing to do with the health economics.

After selection and removal of duplicates, 1,204 documents were retained, and countries, authors, journals, affiliations, and keywords of those documents were subsequently analyzed.

Bibliometrix package (version 3.2.1) in R software (version 4.1.3) was used to analyze the documents. SCImago Graphica Beta (version 1.0.23) was used to present the networks of co-authorship of countries and main authors. The network map, and the density map for keywords were created using VOSviewer WPS Office (version 11.1.0.13703). CiteSpace (version 6.1.R6) was then used to create the figure of keywords bursts. Data aggregation and analysis were conducted in WPS Office (version 11.1.0.13703), the pie chart of document types was generated by OriginPro (Version 2023 OriginLab Corporation, Northampton, MA, USA), and the remaining figures were drawn with Ggplot2 package (version 3.3.5) in R software (version 4.1.3).

## Results

### Document types and publication outputs

**FIGURE 2:**
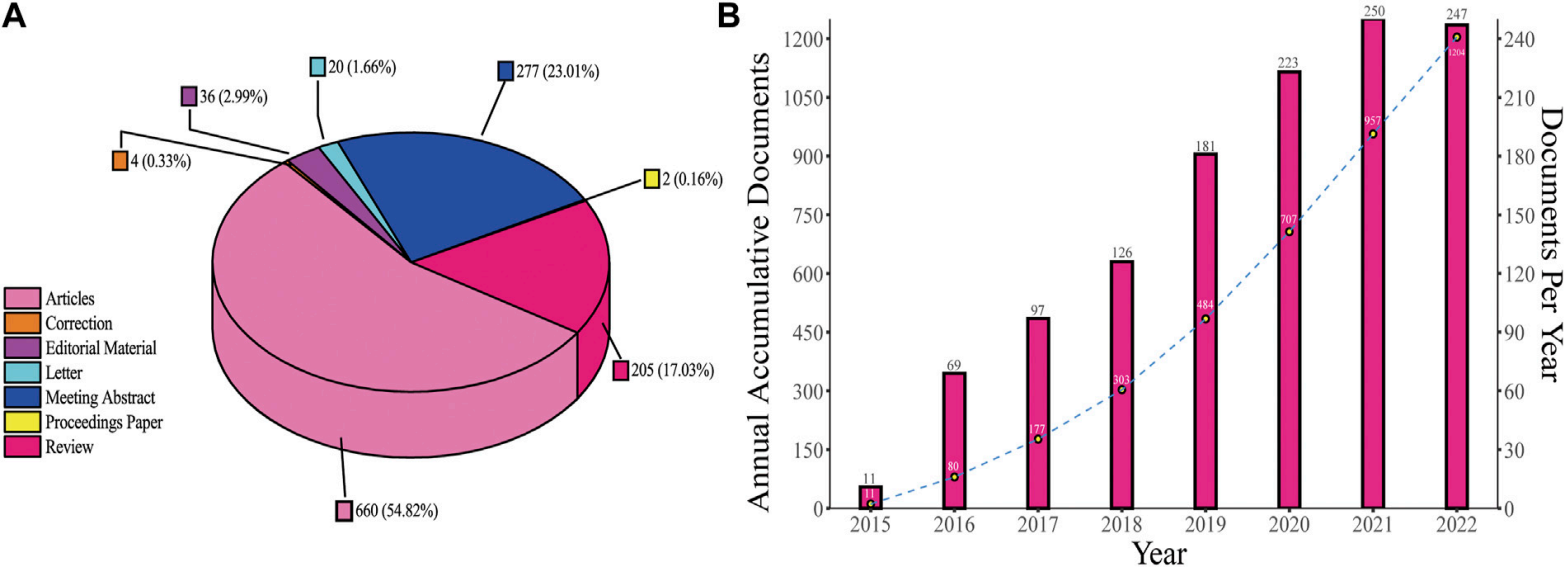
Document types and publication outputs. **(A)** Percentage of each type of document. **(B)** Publications number per year and annual accumulative number.

A total of 1,204 records were selected for inclusion in the study and were divided into seven categories (Figure 2A). Articles accounted for the largest part (54.82%), followed by meeting abstracts (23.01%) and reviews (17.03%), which are close in number. Figure 2B shows the publication output trends from 2015 to 2022. The first study was published in 2015, and since then, the number of publications continuously increased to the year 2021. However, the number of documents in 2022 was almost the same as that in 2021; there were only three fewer publications in 2022 compared with 2021. Annual outputs were less than 100 in the first three years. After 2018, the number of annual publications showed more significant growth, gradually increasing from 126 in 2018 to the maximum of 250 in 2021. From 2018 to 2021, a total of 780 documents were published, accounting for 64.78% of all the documents included in the study.

### Publication distribution of countries, institutes, and authors

**FIGURE 3:**
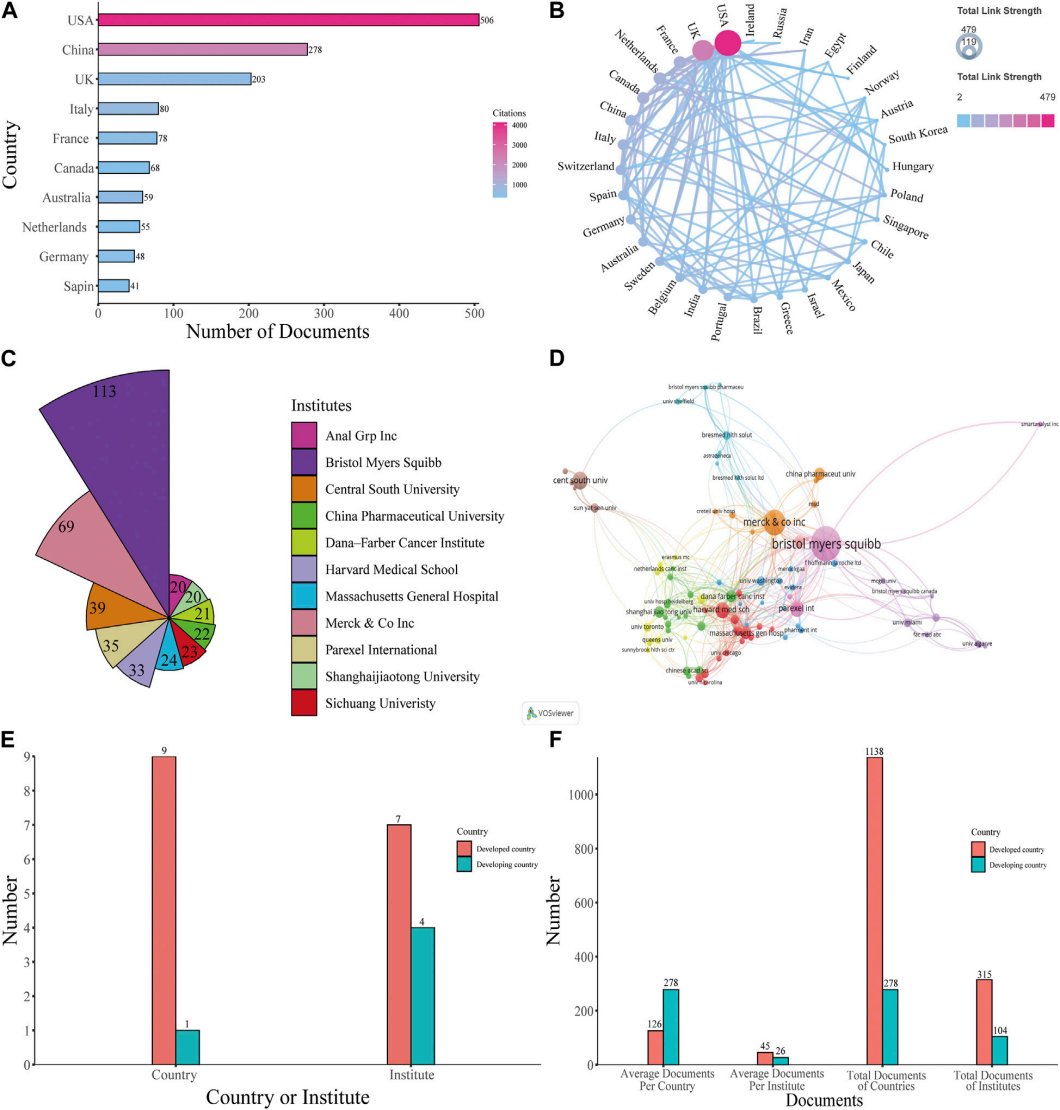
Analysis of countries and institutes. **(A)** The number of articles and citations among the 10 most productive countries with the number of total papers and total citations. **(B)** Filled map of the cooperation between various countries. The color reflects the total link strengths of different countries, while the arrow demonstrates cooperation between the corresponding two countries. **(C)** The top 11 institutes with the most contributions. **(D)** Visualized map of co-authorship of the institutes. **(E)** The number of developed and developing countries among the top 10 productive countries, and the number of institutes from developed and developing countries among the top 11 productive institutes. **(F)** The total number and average number of developed countries and developing countries among the top 10 productive countries, the total number and average number of documents of institutes from developed countries and developing countries among the top productive institutes.

The publications included in the study originated from 64 countries. The United States published the highest number of papers at 506, accounting for 42.03% of all papers, and had the highest number of total citations (4,119). This was followed by China (278 papers, 23.09%, 2,252 citations) and the United Kingdom (203 papers, 16.86%, 597 citations), while other countries with less than or equal to 80 publications (Figure 3A). The cooperation between various countries is reflected in the filled map in Figure 3B. Cooperation partnerships among different countries were strong. The United States and the United Kingdom were the top two countries that had the most cooperation with other countries.

Among the 1850 institutes around the world that were responsible for the selected publication, Bristol Myers Squibb ranked first, with 113 published papers, accounting for 9.39% of all publications. Merck and Co., Inc. was the second institute (69 papers, 5.73%), followed by Central South University (39 papers, 3.24%), Parexel International (35 papers, 2.91%), Harvard Medical School (33 papers, 2.74%), and then other institutes that published less than 30 papers (Figure 3C). Anal Grp Inc. and Shanghaijiaotong University both published 20 papers, so tied in tenth place. Collaborative relationships between diverse institutes with more than 5 published papers are shown in Figure 3D. The results indicated close cooperation between global institutes.

As shown in Figure 3E, most countries among the top 10 productive countries are developed countries and most institutes among the top 11 productive institutes are in developed countries. To better reflect difference among developed countries and developing countries, we made Figure 3F. Among the top 10 productive countries, Developed countries produced significantly more documents compared with developing countries, but on average, a developing country own more documents than a developed country does. For institutes, institutions in developed countries produce more documents, both in terms of total and average publications, than institutes in developing countries do.

### Analysis of authors

**FIGURE 4:**
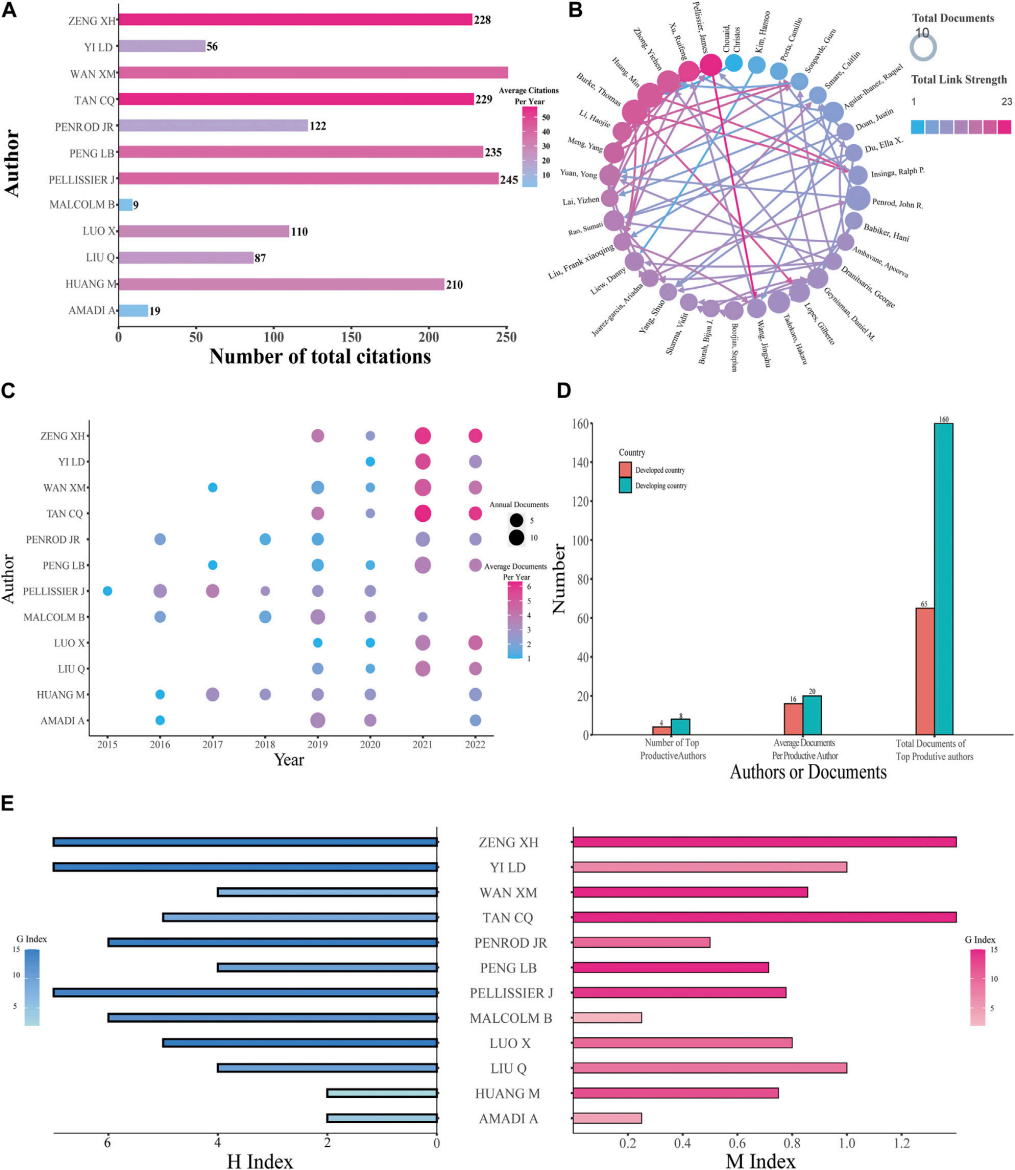
Analysis of authors. **(A)** Total citations and annual citations of the top 12 productive authors. **(B)** The co-authorship of productive authors. **(C)** Annual publications and annual average publications of the top 12 productive authors. **(D)** Number of authors and their documents among the top 12 productive authors. **(E)** G-index, H-index, and M-index of the top 12 productive authors.

In total, 6,033 authors were involved in the study, and the 12 most productive authors were analyzed (the final three authors were tied for tenth place). The United States and China were the main countries with the largest number of authors. Among the most productive authors, the one who has the most citations is Wan XM (Figure 4A). For cooperation of authors with more than five published papers, Pellissier J has a total link strength of 23, ranking first, followed by Xu RF (a total link strength of 21) (Figure 4B). The top three productive authors all came from China; Wan XM published the highest number of papers at 25, while Tan CQ and Zeng XH both ranked second with 24 papers (Figure 4C). The developing countries contain two-thirds of the top 12 productive authors and own 71.11% of the entire documents of the top 12 productive authors. In addition, among the 12 productive authors, developing countries have four more articles per author compared with developed countries (Figure 4D). To analyze authors’ productivity more comprehensively, H-index, M-index, and G-index were used (Figure 4E). Papers from Pellissier J, Tan CQ, and Zeng XH had the highest H-index 7). The G-index of papers from Peng LB, Tan CQ, Wan XM, and Zeng XH was 15, sharing first position. For the M-index of papers, Tan CQ (1.4) and Zeng XH (1.4) both ranked first, followed by Liu Q (1.0) and Yi LD (1.0). Tan CQ and Zeng XH have identical values in these three different indexes. More information about the top productive authors including the number of publications and their corresponding H-index, M-index, and G-index is encompassed in Table 1.

**TABLE 1:** More details of top 12 most productive authors for the research (n%).

Rank	Author	Country	N (%)	H-index	G-index	M-index
1	Wan, XM	China	25 (2.08%)	6	15	0.86
2	Tan, CQ	China	24 (1.99%)	7	15	1.4
3	Zeng, XH	China	24 (1.99%)	7	15	1.4
4	Peng, LB	China	22 (1.83%)	5	15	0.71
5	Luo, X	China	18 (1.50%)	4	10	0.8
6	Huang, M	China	17 (1.42%)	6	12	0.75
7	Penrod, JR	United States	17 (1.42%)	4	10	0.5
8	Liu, Q	China	16 (1.33%)	5	9	1
9	Malcolm, B	Australia	16 (1.33%)	2	2	0.25
10	Amadi, A	United Kingdom	15 (1.25%)	2	4	0.25
11	Pellissier, J	United States	15 (1.25%)	7	14	0.78
12	Yi, LD	China	15 (1.25%)	4	7	1

N (%), the number of published documents of every journal and the percentages show their proportions of total 1,201 documents.

### Distribution of journals, co-cited journals, and co-cited references

**FIGURE 5:**
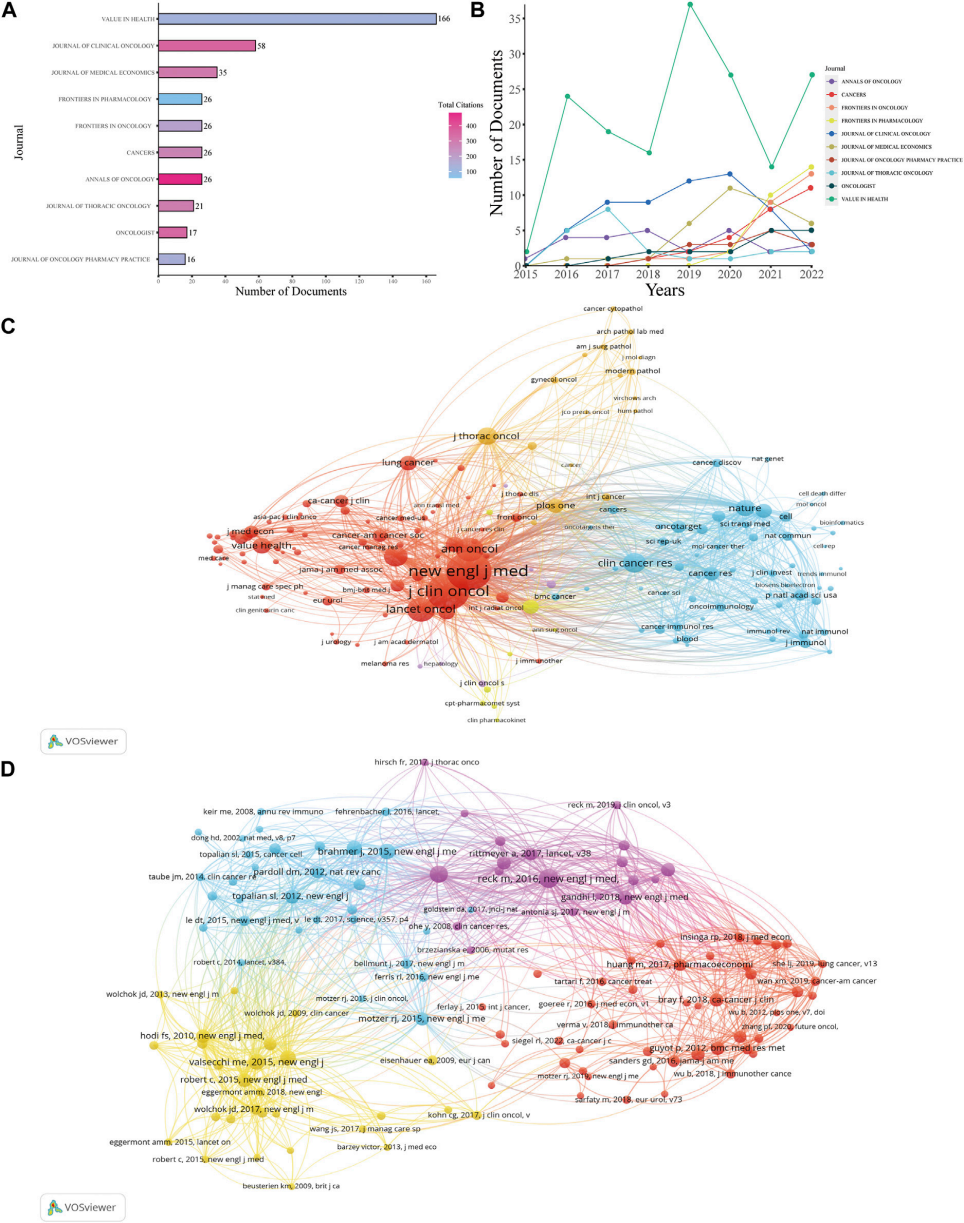
Analysis of journals and references. **(A)** Number of total documents and citations of the top 10 productive journals. **(B)** Number of documents produced annually by the top 10 productive journals. **(C)** Network map of co-cited journals. **(D)** Network map of co-cited references.

Research on the health economics of PD-1 inhibitors and PD-L1 inhibitors has been published in 362 journals. The journal with the most published papers was *Value in Health*, which published 166 papers, accounting for 13.79% of the total papers included in the study (Figure 5A), although this journal only had 132 citations in the papers included in the study. This was followed by *Journal of Clinical Oncology* with 58 papers and 357 total citations in the selected journals. Half of the top 10 journals were in the United States, while the United Kingdom had two journals and Switzerland had three journals. The numbers of annual documents are shown in Figure 5B. More detailed information is provided in Supplementary Table S2.

Figure 5C presents the co-citation map of various journals. *New England Journal of Medicine* had 2,890 co-citations, ranking first, while *Journal of Clinical Medicine* (2,562 co-citations) and *Lancet Oncology* (1,151 co-citations) ranked second and third, respectively. A summary of the top 10 co-cited references according to the total number of citations is presented in Table 2, and Figure 5D is the map of co-cited references that had more than 30 citations, which can directly reflect researchers’ attention according to the number of citations. Two references in the map had been co-cited more than 100 times, including papers published by Reck, Martin et al. (2016), Borghaei, H et al. (2015).

**TABLE 2:** The top cited references and citations.

Rank	Cited references	Citations
1	Reck M, et al., 2016, Pembrolizumab *versus* Chemotherapy for PD-L1-Positive Non-Small-Cell Lung Cancer. N Engl J Med.	142
2	Borghaei H, et al., 2015, Nivolumab *versus* Docetaxel in Advanced Non-squamous Non-Small-Cell Lung Cancer. N Engl J Med.	118
3	Brahmer J, et al., 2015, Nivolumab *versus* Docetaxel in Advanced Squamous-Cell Non-Small-Cell Lung Cancer. N Engl J Med.	95
4	Valsecchi Me, et al., 2015, Combined Nivolumab and Ipilimumab or Monotherapy in Untreated Melanoma REPLY.	95
5	Robert C, et al., 2015, Pembrolizumab *versus* Ipilimumab in Advanced Melanoma. N Engl J Med.	89
6	Garon EB, et al., 2015, Pembrolizumab for the treatment of non-small-cell lung cancer. N Engl J Med.	84
7	Robert C, et al., 2015, Nivolumab in previously untreated melanoma without BRAF mutation. N Engl J Med.	80
8	Gandhi L, et al., 2018, Pembrolizumab plus Chemotherapy in Metastatic Non-Small-Cell Lung Cancer. N Engl J Med.	80
9	Topalian SL, et al., 2012, Safety, activity, and immune correlates of anti-PD-1 antibody in cancer. N Engl J Med.	76
10	Herbst RS, et al., 2016, Pembrolizumab *versus* docetaxel for previously treated, PD-L1-positive, advanced non-small-cell lung cancer (KEYNOTE-010): a randomised controlled trial. Lancet.	73

### Co-occurrence keywords analysis

**FIGURE 6:**
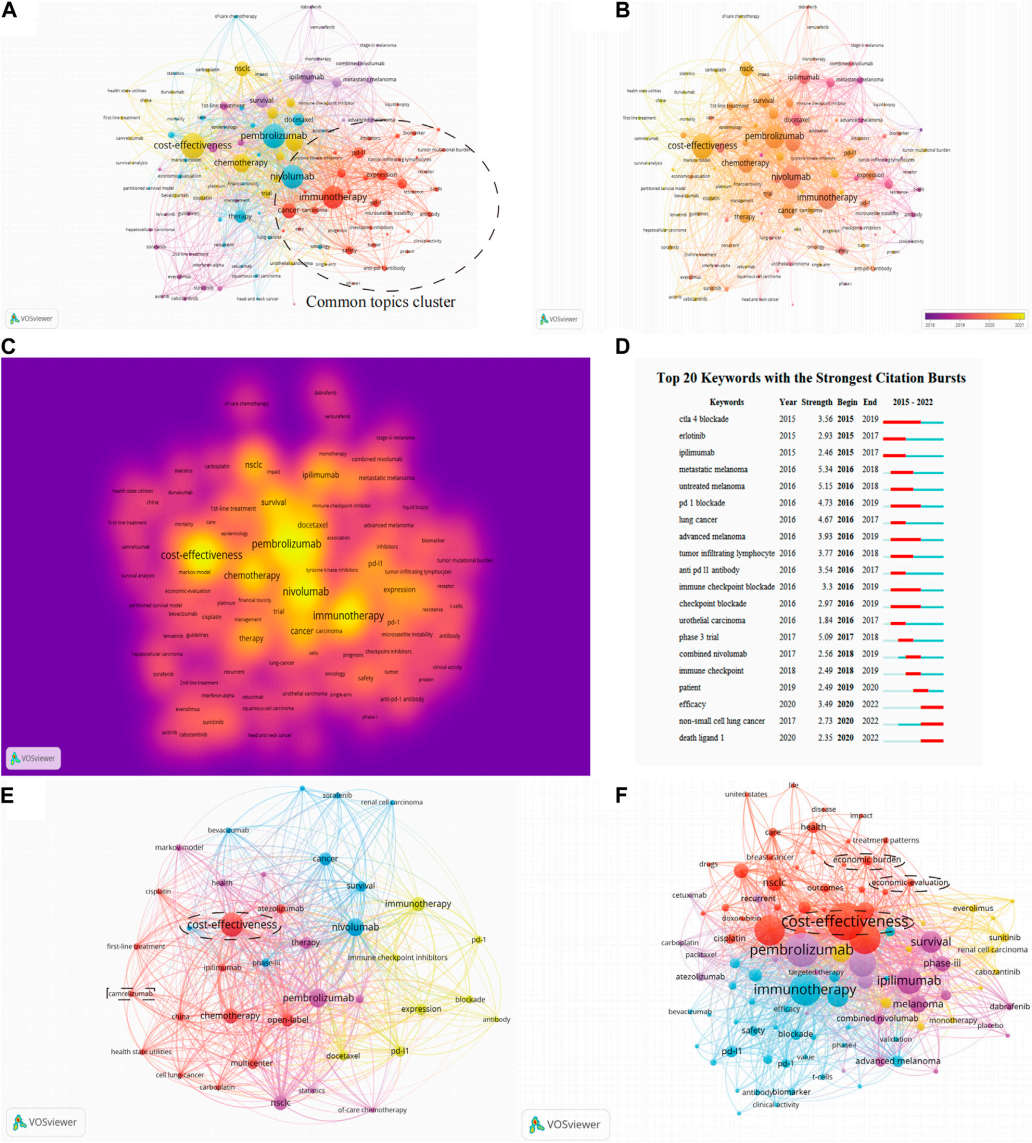
Analysis of keywords. **(A)** Clusters network visualization map of keywords. **(B)** Overlay visualization map of keywords. **(C)** Destiny visualization map of keywords. **(D)** The strongest citation bursts. **(E)** Network visualization map of keywords of documents from China. **(F)** Network visualization map of keywords of documents from the United States.

The study selected 120 keywords that had no less than 10 occurrences. These words include cost-effectiveness of therapy, different drugs, therapy mechanisms, and specific cancers treated with immunotherapy. The keywords with red nodes were grouped into one category in Figure 6A. Keywords with red nodes have a strong connection with nodes of other colors, which means most documents focused their research on keywords with red nodes. Thus, we put keywords with red nodes into the cluster named “Common topics cluster”. This cluster predominantly relates to immunotherapy and its mechanism and includes the key terms “PD-1”, “PD-L1”, “inhibitors”, “cancer”, and “biomarker”. The remaining keywords indicate the health economics study in following three aspects: 1) in different kinds of drugs that are widely used in immunotherapy and chemotherapy; 2) in different periods of clinical treatments; and 3) in more specific cancers, such as melanoma.

Figure 6B shows how the frequency of keywords changes with time. Most keywords have emerged from 2018, which may be related to the awarding of the Nobel Prize in Physiology or Medicine in 2018 for work on PD-1 as a target in cancer therapy. The keywords with the highest density are pembrolizumab, cost-effectiveness, nivolumab, immunotherapy, and chemotherapy. The importance and frequency of different keywords is demonstrated by the destiny visualization map (Figure 6C).

### Burst analysis of keywords

According to Figure 6D, during the early years, the hot topics of this field concerned melanoma and some typical anti-cancer drugs. This may indicate that the health economics study of inhibitors of PD-1 and PD-L1 for the treatment of melanoma had been extensively investigated, while relevant research on other kinds of tumor was not sufficiently mature at that time. During the intervening years, the hot topics were predominantly about the mechanism of immunotherapy with PD-1. In recent years, the emergence of keywords such as “efficacy” and “combined Nivolumab” suggests that research on boosting the effectiveness of PD-1 inhibitor therapy is becoming popular.

Network visualization maps of keywords of documents from China (Figure 6E) and documents from the United States (Figure 6F) were created. The research in developing countries is more in-depth than it in developed countries

## Discussion

This study presented a bibliometric and visualized analysis to summarize the current research status and predict promising research fields of PD-1/PD-L1 inhibitors. To the best of our best knowledge, this is the first study to systematically evaluate this field in regard to contributing countries, institutions, journals, authors, and research hot spots. Moreover, substantial differences between developed and developing countries were revealed. This study provides information that can accurately guide scholars, governments, and pharmaceutical companies on relative study, policy making, and promotion of PD-1/PD-L1 inhibitors.

Research on PD-1/PD-L1 inhibitors in the health economics field is in an uneven situation currently. From Figure 3, the total numbers of documents from different countries and different institutes and the frequency of cooperation between different countries all reflect the unbalanced development in the health economics field of PD-1/PD-L1 inhibitors. According to the analysis of authors in Figure 4, it is obvious how lopsided the productivity and influence of different authors are. Figure 5 demonstrates the discrepancy between different journals; some influential journals published more documents, over 30 documents per year in some cases. In summary, there is markedly uneven development in the research of PD-1/PD-L1 inhibitors in the health economics field in terms of countries, institutes, authors, and journals. Furthermore, the uneven development of the relative research may originate from the gap between the developed countries and the developing countries. Thus, to better identify the reason behind this situation, it is essential to analyze how the differences of the developed and developing countries are formed.

Among all the countries selected in the study, the United States and China are top among the developed and the developing countries, respectively. Consequently, the network visualization maps of keywords of documents from these two countries were analyzed to identify the potential differences and the future trends in the health economics field of PD-1/PD-L1 inhibitors for developed and developing countries. The circle clusters in Figures 6E, F highlight the research hot spots in the health economics field of PD-1/PD-L1 inhibitors for each of the two countries. The hot spots of the United States are more detailed than those of China; besides cost-effectiveness, the United States has gradually started to analyze more specific problems including the economic burden (Nesline et al., 2020; Morgans et al., 2021). This suggests that researchers need to be more precise in their research issues when conducting studies in the health economics field of PD-1/PD-L1 inhibitors if they want to make more progress and have more outputs in the future. Additionally, to elucidate how governments influence the research in the health economics field of PD-1/PD-L1 inhibitors, several factors affecting the new drug marketing process have to be compared (Lanthier et al., 2013; Eder et al., 2014; Darrow et al., 2020; Wang et al., 2022), and the results of this analysis are listed in Table 3. Here are the results after comparing strategies in the United States and China (Center For Drug Evaluation, NMPA, 2022; National Medical Products Administration, 2022; U.S. Food & Drug Administration, 2022; U.S. Department of Health & Human Services, 2022). The application process of new drugs is more efficient in the United States, probably because the United States puts more manpower into examination and approval for marketing. China has relatively strict requirements on the process of data submitted by pharmaceutical companies, while the United States has additional requirements in the quality and comprehensiveness of the clinical research. Furthermore, compared with the United States, China, the prices of drugs in China were generally lower, including Pembrolizumab, Nivolumab, Atezolizumab and Durvalumab. In addition, among these four drugs, Pembrolizumab, Nivolumab and Atezolizumabwhich in China were almost half the prices than in the United States (Huang et al., 2022). To summarize, compared with the United States, the marketing process of new drugs in China is more conservative. Moreover, in both countries, the comprehensive coverage of medical care has certain influencing factors in the marketing of PD-1/PD-L1 inhibitors. Overall, for the governments, if they want to propel the research in the health economics field of PD-1/PD-L1 inhibitors, they can: 1) accelerate to include the relevant drugs in national insurance so that these inhibitors can be more affordable, thereby expanding the markets for PD-1/PD-L1 inhibitors; 2) optimize the approval process for new drugs to reduce unnecessary time and effort; and 3) solve the lack of talented people in the field of new drugs approval process as soon as possible to improve the efficiency of the approval process.

**TABLE 3:** Factors affecting the new drugs marketing process.

Item	United States (developed country)	China (developing country)
Review procedure and application process	One declaration and direct review	Repeated declarations and hierarchical approval
Number of approved anticancer drugs in China and the United States from 2012 to 2021	118	87
Price information of drugs in China and the United States	Pembrolizumab: $ 5,102	Pembrolizumab: $ 2,818
	:Nivolumab: $ 3,697	:Nivolumab: $ 1,456
	Atezolizumab: $ 9,411	Atezolizumab: $ 5,158
	Durvalumab: $ 3,697	Durvalumab: $ 2,844
Policy to accelerate process	PR, AA, FTD, BTD	PR, BTD, CA, SRA
Insurance coverage in 2022	92%	>95%

PR: Priority Review; AA: Accelerated Approval; FTD: Fast Track Designation; BTD: Breakthrough Therapy Designation; CM: Conditional Approval; SRA: Special Review and Approval.

Besides the differences between the developed countries and the developing countries, the analysis also revealed information that would be helpful for developing countries. The rectangular box in Figure 6E reveals the current possible development trend of the developing countries in the health economics field of PD-1/PD-L1 inhibitors. Camrelizumab is the third drug officially developed and successfully marketed in China. In terms of marketing time, this drug is not as advantageous as the previous two domestic drugs, however, the health economics research of this kind of drug is more than that of the previous two domestic drugs. According to those previous bibliometric analyses, there are two options for a country like China that were relatively late in developing the research in this field: 1) focus on other kinds of chemical drugs for co-treatment with PD-1/PD-L1 inhibitors to improve the efficacy of inhibitors; 2) continue to develop novel PD-1/PD-L1 inhibitors and attempt to enter the overseas markets. Clearly, the first option far exceeds the second option in terms of economic benefits. Excessive research in the development of PD-1/PD-L1 inhibitors may cause wastage of clinical materials and scientific research funds after prolonged research without results. Furthermore, the drug market is highly competitive given the global economic downturn in recent years (The World Bank, 2022). However, the bibliometric analysis of the health economics of Camrelizumab has already revealed that even with the impact of the global great economic downturn, for countries that are still developing in this field, the second option—that is, applying money, manpower, and time for the development of new drugs—is a possible but necessary path.

Our results reveal the potential trends of the health economics field of PD-1 inhibitors and PD-L1 inhibitors. The bursts words shows that “phase III trial” emerged in 2017, which means clinical trials of these two inhibitors have appeared for at least five years. Furthermore, the overlay visualization map of the keywords also contains some noticeable labels including “Docetaxel”, “Cisplatin”, “Dabrafenib”, and “Ipilimumab”, and although none of these drugs are PD-1 inhibitors or PD-L1 inhibitors, their emergence is related to the shift of the research hot spot. In the last few years, much related research of this health economics field has focused on the comparison of the curative effect of PD-1 inhibitors, PD-L1 inhibitors, and other kinds of chemical drugs. However, since 2018, the proportion of clinical trials of single-drug immunotherapy has begun to decrease and is being replaced by combined immunotherapy, which uses both PD-1/PD-L1 inhibitors and other types of chemical drugs to increase the curative effect of the immunotherapy. Meanwhile, not only are combination therapy trials accounting for a large part of the research, but drug combinations are also shifting from using chemotherapy and anti-CTLA4 combinations to other targeted methods to bypass drug resistance mechanisms and thus achieve greater efficacy of PD-1/PD-L1 inhibitors.

In 2022, the number of published documents in the health economics field of PD-1 inhibitors and PD-L1 inhibitors stopped increasing, which indicates saturation of the research areas. For the researchers who continue to study in this field, we suggest a number of further analyses for the future: 1) a more specific comparison of analysis of the health economics of different kinds of PD-1 inhibitors and PD-L1 inhibitors, such as Nivolumab and Pembrolizumab. This kind of comparison can help to demonstrate the economic development of different kinds of PD-1 inhibitors and PD-L1 inhibitors and provide more detailed information for future research in the health economics of PD-1/PD-L1 inhibitors; 2) developing appropriate biomarkers to increase the precision when using targeted and differentiated PD-1/PD-L1 inhibitors; 3) exploration of the economic benefits of PD-1/PD-L1 inhibitors in other cancers that have not been studied previously; 4) a comparison of research output trends in the health economics of other kinds of drugs that are widely used for immunotherapy, which may help to explain some of the situations that have occurred in the health economics field of PD-1 inhibitors and PD-L1 inhibitors; 5) to confirm the optimal dose of PD-1 inhibitors and PD-L1 inhibitors for treatment; and 6) periodic repetition of such analyses to observe the potential trends in research outputs and finally to encourage collaborations of different scientific research teams.

Similar to all other bibliometric analyses (Gao et al., 2019; Liu et al., 2022), there are limitations in our study. Final results of the study are affected by the choice of database (Web of Science Core Collection) and the search queries used. The selected documents included a few articles that focused on the efficacy of PD-1 inhibitors but pointed out that their conclusions were helpful to the study of health economics, and this is where the major limitation lies. This also emphasizes the need not only for skill in data searches and analysis, but also for expertise in the selected field to ensure the accuracy of the criteria for the process of selecting documents.

This study found that the United States led in this research field by contributing 42.03% of the total publications. The top productive journal is *Value in Health*. The top co-cited journal is *New England Journal of Medicine*, and active collaborations among countries and institutes can be observed. The top productive institute is Bristol Myers Squibb, which is in the United States. The most productive author is Wan XM. For the global trend of this field, the cost effectiveness of PD-1/PD-L1 inhibitors and comparative analysis of the economic effects of PD-1/PD-L1 inhibitors and other drugs are the predominant hot topics. For pharmaceutical companies that are developing PD-1/PD-L1 inhibitors, we suggest they should exploit overseas markets. Finally, for developing countries who continue to study the health economics field of the PD-1/PD-L1 inhibitors, the expansion of medical insurance coverage and acceleration of the marketing process of new drugs are needed.

## Data Availability

The raw data supporting the conclusion of this article will be made available by the authors, without undue reservation.
